# Griffin Auxiliary Medical Society

**Published:** 1859-04

**Authors:** 


### GRIFFIN AUXILIARY MEDICAL SOCIETY.

We are gratified to learn that the physicians of Griffin and surrounding country, have organized a Medical Society to be known as the "Griffin Auxiliary Medical Society."

The objects and advantages of such societies is eloquently portrayed by Dr. E. F. Knott, of Griffin, Ga., in an address delivered at the organization of the above society, and to be found in the original department of the present number of this journal. We do not now recollect when we have read an ·ddress with more pleasure, or been more encouraged to do battle for that science which we so much love. We do feel, that if every physician in our State, would read carefully this address, that much good would result to our noble profession.

But few who have never been members of county or local medical societies, would be able to calculate the great good that they may be made to accomplish. Aside from a mutual interchange of opinions upon scientific and practical subjects, resulting in good, both to science and each member of the society, much is done to cultivate that fraternal feeling, which is so desirable among members of our profession, and to extinguish the last spark of that jealousy which sometimes prompts one brother to seek an advantage of another, to the injury of both, and that noble science which all must admire.

From our intimate acquaintance with a number of the gentlemen composing this body, we feel confident that it will not "die out" as we have known some to do, but will constantly grow in interest.

